# Sociophysics Analysis of Multi-Group Conflicts

**DOI:** 10.3390/e22020214

**Published:** 2020-02-14

**Authors:** Miron Kaufman, Hung T. Diep, Sanda Kaufman

**Affiliations:** 1 Department of Physics, Cleveland State University, Cleveland, OH 44115, USA; 2 Laboratoire de Physique Théorique et Modélisation Université de Cergy-Pontoise, CNRS, UMR 8089, 2 Avenue Adolphe Chauvin, 95302 Cergy-Pontoise CEDEX, France; diep@u-cergy.fr; 3 Levin College of Urban Affairs, Cleveland State University, Cleveland, OH 44115, USA; s.kaufman@csuohio.edu

**Keywords:** social conflicts, statistical physics approach, complex systems, mean-field theory, monte carlo simulation

## Abstract

We present our research on the application of statistical physics techniques to multi-group social conflicts. We identify real conflict situations of which the characteristics correspond to the model. We offer realistic assumptions about conflict behaviors that get factored into model-generated scenarios. The scenarios can inform conflict research and strategies for conflict management. We discuss model applications to two- and three-group conflicts. We identify chaotic time evolution of mean attitudes and the occurrence of strange attractors. We examine the role that the range of interactions plays with respect to the occurrence of chaotic behavior.

## 1. Introduction

Social conflicts have been subject of investigations for decades [1,2,3,4,5]. Numerous approaches and methods to study social conflicts have been proposed by several disciplines [6,7,8], including statistical physics. The relevance of statistical physics tools to social phenomena, which has been called sociophysics, has been debated [9,10]. It consists of finding analogies between individual and group social interactions and physics concepts, allowing the application of physics models to social phenomena. For other physics-inspired studies of conflict, from the perspective of making and sustaining peace, see References [11,12,13]. The interested reader can consult these papers for earlier references to this type of research.

The parties to social conflicts operate in contexts of constantly shifting complex social, political, and economic circumstances that affect their decisions. Therefore, each group seeks to formulate a strategy for reaching beneficial outcomes and for mitigating negative ones. However, at eye level, this requires well-developed theories or causal mental models that link specific decisions to outcomes, considerable amounts of information in real time, and multi-party decision processes. These strategy ingredients are often unavailable or incomplete and are difficult to assemble, hampering groups’ abilities to work toward specific desired outcomes. Statistical physics can help capture and study some key aspects of the inter-group interactions and to generate ranges of outcomes that groups can use to anticipate conflict trajectories and ranges of outcomes and to formulate strategies despite a lack of prediction ability which is limited in complex systems.

Statistical physics studies large systems of interacting particles using their microscopic properties [14] to predict macroscopic properties. Spin models from statistical physics have been used to investigate behaviors of social systems, which are composed of a large number of individuals who interact with each other in a manner akin to agitation which can be associated with temperature. These models have been used to study complex social systems such as culture dynamics, crowd behavior, information dissemination, social conflict, and economic matters. One critique leveled at the sociophysics approach is that it tends to oversimplify social dynamics and the diversity of individual characteristics that matter [15]. However, we see behavior regularities in opinion surveys despite individual uniqueness. This observation strengthens our belief that the use of statistical physics to model social conflict is appropriate [16,17]. Averaging over large societal groups washes away individual particularities while retaining shared characteristics that affect conflict outcomes. We have used this approach to analyze concrete situations such as the Brexit referendum [18,19], the US election in 2016 [18,19,20,21], and the Serbia–Herzegovina–Croatia election in 2018 [22,23]. We have generated anticipatory scenarios for these conflicts. Note that spin models have also been used to study the price variation in stock market exchange [24].

Methods in statistical physics include numerical simulations. With an extremely rapid increase of computer capacities in speed and memory, simulations have become an efficient means to study complicated systems where analytical approximations encounter much difficulties. Monte Carlo simulations are very useful in many domains, in particular in statistical physics [14,25]. This method has been used with success for sociophysics [18,22,26] and econophysics [24].

In what follows, we present our research on the sociophysics of conflicts that yielded a model of multi-group interactions. We apply statistical physics concepts and techniques to model multi-group interactions such as those occurring in national and international settings. Both mean-field theory and Monte Carlo simulation are used.

We first considered that every member of each group interacts with each other member through persuasion to alter each other’s attitude toward a specific conflict. Using Monte Carlo simulations, we explored effects of the network topology on the qualitative behavior of this two-group model. The model predictions include temporal oscillations of the attitudes towards negotiation or continued stalemate. The Monte Carlo simulations also exhibit chaotic time dependence of the mean attitudes. Using this model, we generated scenarios for the two-group conflicts surrounding the Brexit referendum and the US elections, both in 2016, and anticipated their outcomes [18]. We extended the model to three-group interactions and used it to generate scenarios of possible outcomes for the 2018 Bosnia and Herzegovina elections [22].

The balance of this paper is organized as follows. In Section 2, we generalize our statistical sociophysics model of inter-group conflicts. In Section 3, we show some of the results from two- and three-group dynamics that include oscillations and chaotic behavior. In Section 4, we present Monte Carlo simulations with short-range interactions between individuals. The concluding remarks in Section 5 propose some future model enhancements.

## 2. Mean-Field Model

We began by considering two disputing groups. In each group, each individual has an attitude, described by a discrete variable *S*, regarding whether and how the conflict should be resolved. In group 1, individuals’ attitudes range from $-M_{1}$ (collaborative, very open to negotiating some agreement) to $M_{1}$ (adversarial, inclined to protracted conflict due to extreme adherence to the group’s position or ideology). Similarly, in group 2, individuals’ attitudes *S* range from $-M_{2}$ to $M_{2}$. In this system, the noise deriving from contextual sources is quantified as a “social temperature” *T*. Individuals interact in time with each other within their own group and with individuals of the other group. The Hamiltonian *H* that describes those interactions depends on the attitudes variables and on four couplings: two intra-group and two inter-group. The two intra-group couplings are not necessarily equal. Unlike physics phenomena which obey Newton’s third law, in the world of humans, the magnitudes of action and reaction are not necessarily equal. We used the Boltzmann probability weight, exp(−H/T), to compute the probability distributions for attitudes.

Each individual interacts with every other individual inside their own group, forming a network of members. This linkage pattern among members of a group based on some shared characteristics is called homophily [27,28], in the words of McPherson et al. [27], “similarity breeds connection”. The networks can interact with each other, forming a multiplex. Each individual in group *n* works with a certain intensity $j_{n}$ to persuade others in his/her own group to his/her point of view and is in turn subject to others’ persuasion efforts. In any group, the individuals’ stances are also affected by the “average” stances of other groups, even if individuals do not necessarily communicate across groups. As group members interact and consider the opposing groups’ attitudes, their own group’s resulting preference average at any time *t* is $S_{n}$ (n=1,2). In each group *n*, an individual’s intensity of advocacy (conceptualized as “negative energy/temperature”) is $j_{n}*S*S_{n}$. The inter-group intensity of interaction resulting from an individual’s consideration of an opposing group’s stance is taken to be proportional to the product between that individual’s preference *S* and the mean value of the preferences of the other groups’ members. For example, for an individual in group 1, this interaction is $k_{12}*S*S_{2}$. Using the Boltzmann distribution, we calculate the average stances in each group:

(1) $$\begin{array}{ccc} S_{1}\left(t+1\right) & = & \frac{\sum_{S=-M_{1}}^{M_{1}}Se^{S[j_{1}S_{1}\left(t\right)+k_{12}S_{2}\left(t\right)]}}{\sum_{S=-M_{1}}^{M_{1}}e^{S[j_{1}S_{1}\left(t\right)+k_{12}S_{2}\left(t\right)]}} \end{array}$$

(2) $$\begin{array}{ccc} S_{2}\left(t+1\right) & = & \frac{\sum_{S=-M_{2}}^{M_{2}}Se^{S[j_{2}S_{2}\left(t\right)+k_{21}S_{1}\left(t\right)]}}{\sum_{S=-M_{2}}^{M_{2}}e^{S[j_{2}S_{2}\left(t\right)+k_{21}S_{1}\left(t\right)]}} \end{array}$$

We introduce a lag time as we assume the preference *S* at time t+1 interacts with the averages $S_{1}$ and $S_{2}$ at an earlier time *t*. The time is measured in units of the delay time. The sums above can be expressed using the Brillouin function [14]:

Equations (1) and (2) can be written as follows:

(3) $$\begin{array}{ccc} S_{1}\left(t+1\right) & = & B(j_{1}S_{1}\left(t\right)+k_{12}S_{2}\left(t\right),M_{1}) \end{array}$$

(4) $$\begin{array}{ccc} S_{2}\left(t+1\right) & = & B(j_{2}S_{2}\left(t\right)+k_{21}S_{1}\left(t\right),M_{2}) \end{array}$$

where the Brillouin function is

(5) $$B\left(\beta ,M\right)=\left(M+\frac{1}{2}\right)\mathrm{cotan}h\left[\left(M+\frac{1}{2}\right)\left(\beta \right)\right]-\frac{1}{2}\mathrm{cotan}h\left[\frac{1}{2}\left(\beta \right)\right]$$

In our dynamic model, the changes in preferences are captured by assuming that the intensity of interactions involves the product of individuals’ preferences at a current time and average preferences of the groups at an earlier time. This lag represents the delay between individuals’ persuasion efforts in one time period and the effects likely to emerge in a later time period. The delay time is the time needed for attempting to change the stance of other members of the group. We have also analyzed three-group conflicts by extending the model. More generally, one can consider *N* groups in conflict [19]. The model equations are as follows:

(6) $$\vec{S(t+1)}=B\left(\overset{↔}{k}*\vec{S(t)},\vec{M}\right)$$

where:

(7) $$\vec{S}=\left(\begin{array}{c} S_{1}\\ S_{2}\\ \vdots \\ S_{N} \end{array}\right),$$

(8) $$\overset{↔}{k}=\left(\begin{array}{ccc} j_{1} & ... & k_{1N}\\ \vdots & \vdots & \vdots \\ k_{N1} & ... & j_{N} \end{array}\right),$$

(9) $$\vec{M}=\left(\begin{array}{c} M_{1}\\ M_{2}\\ \vdots \\ M_{N} \end{array}\right),$$

The interaction matrix *k* has on the diagonal the intra-group interactions *j* that determine the cohesiveness of each group, and the off-axis terms are the inter-group interactions.

(10) $$S_{n}\left(t+1\right)=B\left(\sum_{i=1}^{N}k_{ni}*S_{i}\left(t\right),M_{n}\right)$$

with $k_{nn}=j_{n}$ for any *n* between 1 and *N*. In view of the individuals agency, the matrix of interaction is not symmetric. All entries in the matrix *k* include a factor of 1/T.

## 3. Results: Oscillations and Chaos

**Figure 1:**
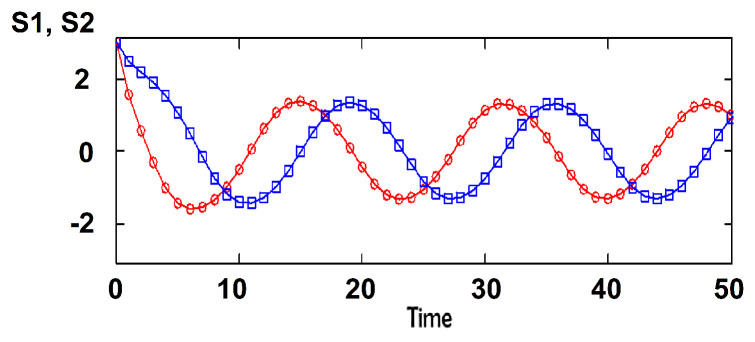
Oscillations in mean attitudes of anti-Brexit (group 1, red) and pro-Brexit (group 2, blue); $j_{1}=j_{2}=0.25$, $k_{12}=-0.1$, $k_{21}=0.1$.

**Figure 2:**
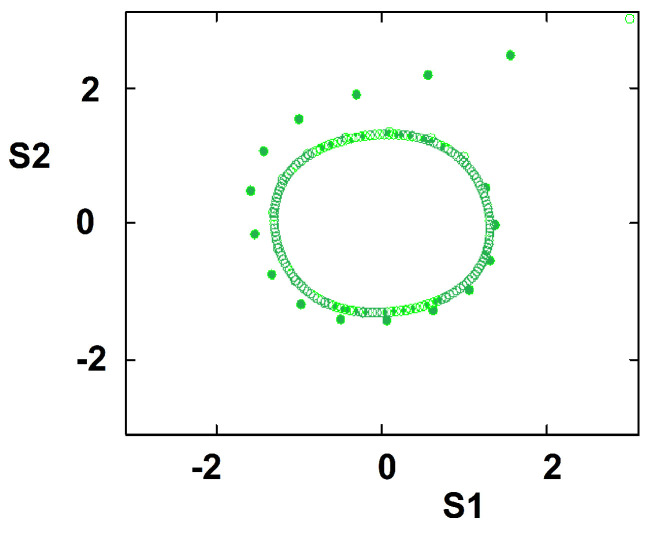
Attractor for $j_{1}=j_{2}=0.25$, $k_{12}=-0.1$, $k_{21}=0.1$. Trajectories started at any initial point converge to the limit cycle.

We have introduced and discussed this model for two groups [18,22]. The number of possible attitudes is the same for all individuals: $M_{1}=M_{2}=3$ corresponding to q=2M+1=7 states for each individual. This choice was intended to parallel typical opinion polls where respondents consider a few options along Likert-type scales. In References [18,22], we have also considered other numbers of states *q*. The stability of the system was considered [18]. By using a linear approximation of the dynamic equations, we identified the regions of the parameter space where the ordered phase can exist along with the disordered phase. An interesting outcome of the model is the occurrence of oscillations in the attitudes’ time dependence when the inter-group interactions have opposite signs; for two groups, $k_{12}*k_{21}<0$. We have argued that this applies to the Brexit conflict inside Great Britain. Group 1 is composed of the supporters of continued membership in the European Union (EU); group 2 contains individuals who want to exit the European Union. The case is characterized by time oscillations, with the pro-EU group and the pro-Brexit group alternating in leading in polls at different times. We chose relatively high values for cohesive interactions $j_{1}$ and $j_{2}$, with $k_{12}<0$ and $k_{21}>0$ to obtain similar oscillations sustained in time. $k_{12}<0$ reflects that the extremists of the pro-Brexit group 2 encourage pro-EU group 1 members to be accommodating, while the compromising wing of group 2 fuels the extremists in group 1. $k_{21}>0$ means that extreme wing of group 1 strengthen the extreme wing of group 2, while the moderate wing of group 1 helps group 2 moderate. The resulting dynamics is shown in Figure 1. When representing the conflict dynamics in the $\left(S_{1},S_{2}\right)$ plane, we get an attractor (limit cycle) for all initial conditions; see Figure 2.

**Figure 3:**
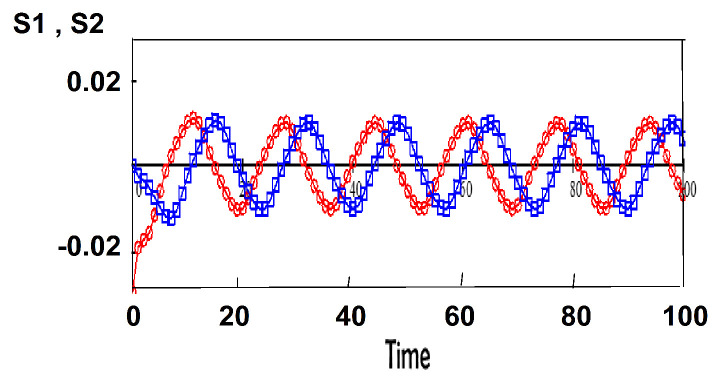
Slightly different initial conditions do not diminish or increase over time. anti-Brexit (group 1, red) and pro-Brexit (group 2, blue); $j_{1}=j_{2}=0.25$, $k_{12}=-0.1$, $k_{21}=0.1$.

If we start from slightly different initial conditions, the difference between the attitudes neither explodes exponentially fast (as one expects for chaos) nor diminishes to zero as one gets for a stable fixed point. The limit cycle implies that the differences between the attitudes starting from different initial conditions oscillate in time, as seen in Figure 3.

**Figure 4:**
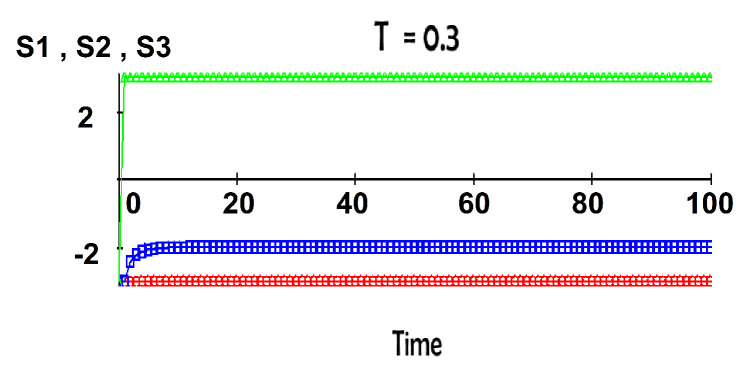
Low temperature T=0.3.

We found no instances of chaotic dynamics for the two-group model. We turned to the study of three-group conflicts. Their dynamics are complex, with fixed points and limit cycles depending on the parameter values. For certain choices of the mutual couplings, the three-group model does exhibit chaotic dynamics. In these cases, the limit cycles are replaced by an attractor with complex geometry. To illustrate these phenomena, we fix the values of parameters *j* and *k* and vary the temperature *T*. The fixed interaction values are $j_{1}=0.2$, $j_{2}=0.3$, $j_{3}=0.1$, $k_{12}=0.5$, $k_{21}=0.5$, $k_{13}=0$, $k_{31}=0$, $k_{23}=0.5$, and $k_{32}=-1$. At low temperatures, the attitudes $S_{1}$, $S_{2}$, and $S_{3}$ approach in time fixed values, as seen in Figure 4.

**Figure 5:**
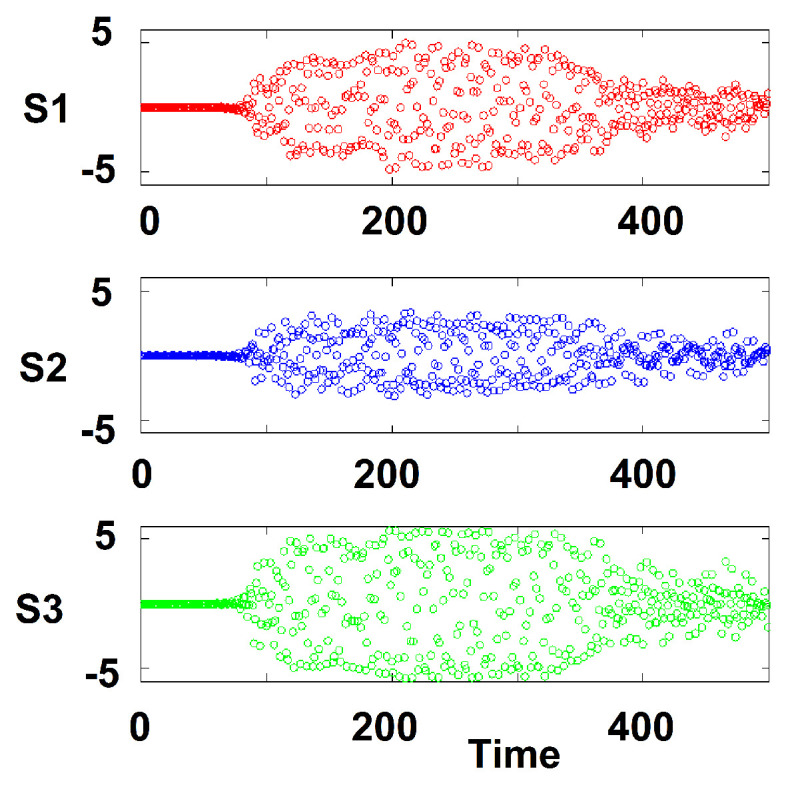
T=1, sensitivity to initial conditions.

At higher temperatures, the dynamics becomes chaotic. In Figure 5 we show the time evolution of attitudes starting from slightly different initial conditions. For a short time, the difference is close to zero and then it explodes in irregular and unpredictable fashion.

**Figure 6:**
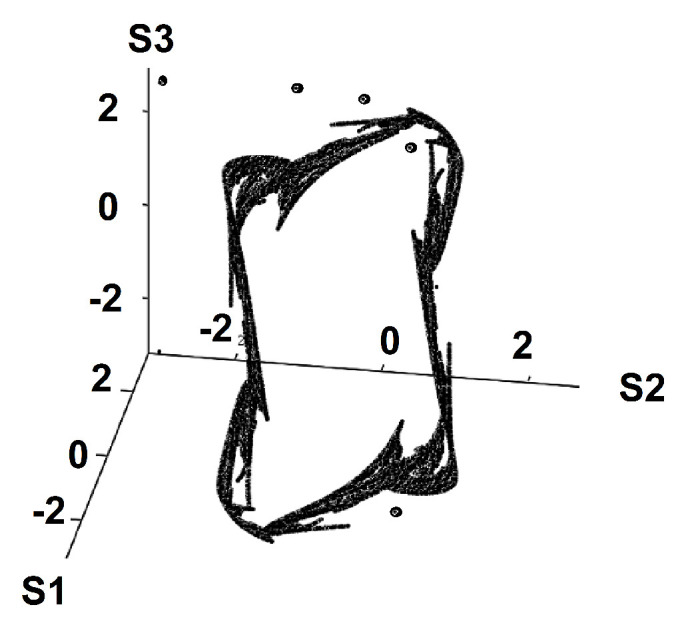
T=1. Strange attractor.

When viewed in the three-dimensional space of attitudes $S_{n}$, the trajectory approaches a strange attractor, see Figure 6. Strange attractors are associated with chaotic dynamics as was shown by Lorenz [29] for a fluid mechanics problem.

**Figure 7:**
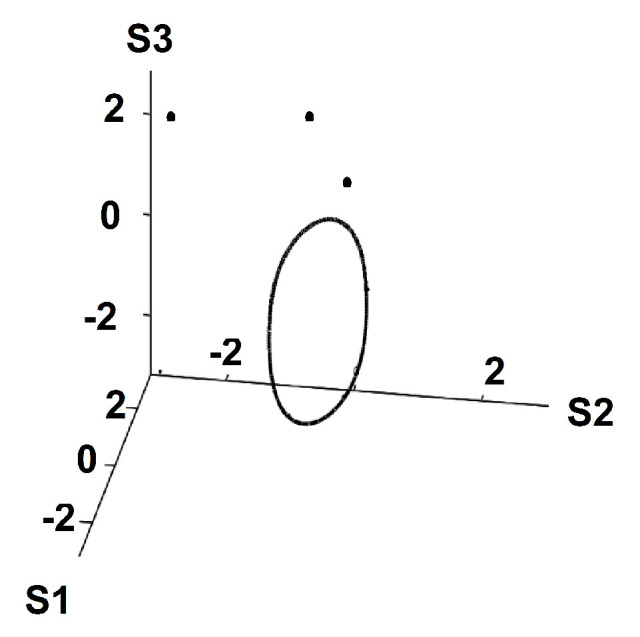
T=2. Limit cycle attractor.

As the temperature is raised further, the strange attractor diminishes in size, see Figure 7, eventually becoming a fixed point at $S_{1}=S_{2}=S_{3}=0$ for T>2.2.

## 4. Monte Carlo Simulations

In this section, we show results obtained by Monte Carlo simulations. The model for simulations is the same as that used in the mean-field theory in the previous section with the same retarded interaction. However, for the simulation, we limit the interaction of an individual in one group at time t+1 to a limited number of neighbors (nearest neighbors) of his/her group in their state at time *t*. This individual interacts with the “average states” of other groups at time *t*. To simplify the presentation, we consider two groups, but this can be generalized straightforwardly to more groups.

We use the Metropolis algorithm for the simulation [25]. We consider two groups of populations $N_{n}$ and $N_{n^{\prime }}$. Each individual has 12 NN of the same group. We take $N_{n}=N_{n^{\prime }}=1600$ which is a typical size in opinion surveys. In canonical Monte Carlo simulations, the social temperature *T* is fixed. We explicitly express the temperature *T* and the interactions from the mean field theory (MF)

(11) $$\begin{array}{ccc} j_{1}\left(MF\right) & = & \frac{J_{1}}{k_{B}T},\;\;\;j_{2}\left(MF\right)=\frac{J_{2}}{k_{B}T} \end{array}$$

(12) $$\begin{array}{ccc} k_{12}\left(MF\right) & = & \frac{K_{12}}{k_{B}T},\;\;\;k_{21}\left(MF\right)=\frac{K_{21}}{k_{B}T}, \end{array}$$

with $k_{B}$ being the Boltzmann constant.

For the case of two groups, the energy of an individual *i* in group *n* at time t+1 is given by

(13) $$E_{n}\left(i,t+1\right)=-J_{n}S_{n}\left(i,t+1\right)\sum_{j}S_{n}\left(j,t\right)-K_{n,n^{\prime }}S_{n}\left(i,t\right)\bar{S_{n^{\prime }}\left(t\right)}$$

where the sum is taken over nearest neighbors (NN) *j* belonging to group *n*, with interaction $J_{n}$. The second term is the interaction with the average of the other group $n^{\prime }$ at *t*:

(14) $$\bar{S_{n^{\prime }}\left(t\right)}=\frac{1}{N_{n^{\prime }}}\sum_{j\in n^{\prime }}S_{n^{\prime }}\left(j,t\right)$$

where $N_{n^{\prime }}$ is the population of group $n^{\prime }$.

**Figure 8:**
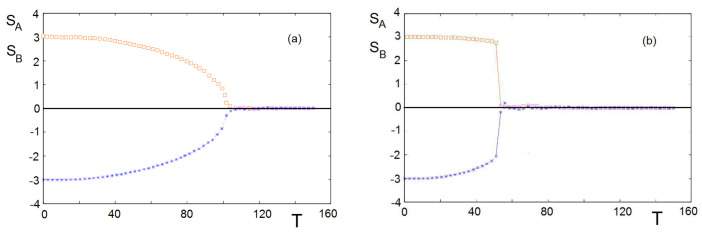
(**a**) No inter-group interaction with $J_{A}=J_{B}=0.02$, $M_{A}=M_{B}=7$ and initial conditions $S_{A}=-S_{B}=3$: the two groups loose their “cohesiveness” above $T_{c}^{0}≃102$ (arbitrary unit); (**b**) with inter-group interaction $-K_{AB}=K_{BA}=0.005$, both groups loose their “cohesiveness” above $T_{c}=53$.

The simulation is carried out as follows. For a given set of interaction $\left(J_{1},J_{2},K_{12},K_{21}\right)$, we generate separately a random state of each group. At a given *T*, we consider first two groups without inter-group interaction. The objective is to determine first the cohesiveness of each independent group as a function of *T*. We take an individual and calculate his/her energy *E*. We change at random his/her state and calculate his/her new energy $E^{\prime }$. If $\Delta E=E^{\prime }-E<0$, then we take his/her new state. If $\Delta E=E^{\prime }-E>0$, we take his/her new state if a random probability *P* (0≤P≤1) is larger than $\exp[-\Delta E/k_{B}T]$. Otherwise, we keep the old state. We repeat this, updating for all individuals of each group for a large number of sweeps to get the equilibrium state for each group. We calculate for varying *T*. The results for an example are shown in Figure 8, where we used $M_{n}$=3, namely 7-state individuals with $S_{n}=-3,-2,-1,0,1,2,3$. Figure 8a shows the cohesive strengths of two independent groups *A* and *B* measured by the value of $|S_{n}|$ (n=A,B). They are equal because we have taken $J_{A}=J_{B}$. Above the critical temperature $T_{c}^{0}≃102$, both of them loose their internal cohesiveness, namely $|S_{n}|=0$. When we turn on the interactions between them with opposite signs, the critical temperature goes down to $T_{c}=53$, as seen in Figure 8b.

**Figure 9:**
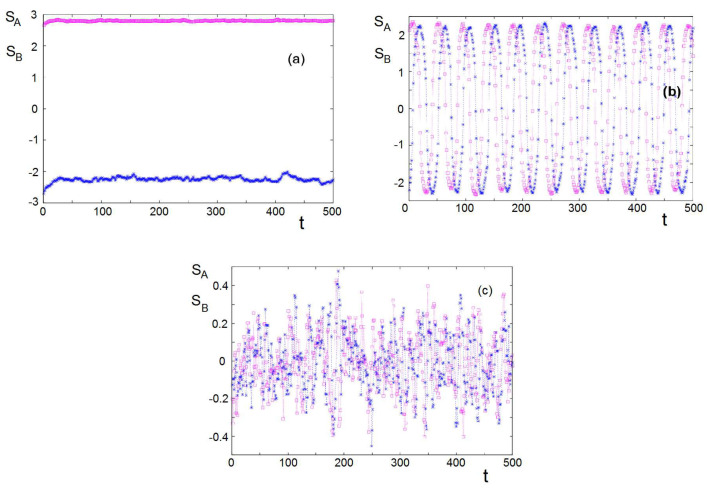
Dynamics observed with $-K_{AB}=K_{BA}=0.005$, $J_{A}=J_{B}=0.02$, $M_{A}=M_{B}=3$, and the initial condition $S_{1}=-S_{2}=3$: (**a**) at “social temperature” T=48 below $T_{c}=53$ where both groups are “ordered”, (**b**) at “social temperature" T=74 between $T_{c}=53$ and $T_{c}^{0}=102$ (cf. Figure 8), and (**c**) at T=125 above $T_{c}^{0}$ where both groups when independent are disordered. See text for comments.

Let us examine the dynamics of the groups at different social temperatures shown in Figure 9. At $T<T_{c}=53$ where both groups are ordered, each keeps its own stance (keeping its sign) as seen in Figure 9a. However, for $T_{c}<T<T_{c}^{0}$, their stances oscillate with time as seen in Figure 9b. This oscillatory behavior has been observed in the mean-field theory shown above. For $T>T_{c}^{0}$, the variation of the stances is uncorrelated and chaotic as seen in Figure 9c. This chaotic behavior was not observed in the two-group conflicts in the mean-field theory. It is due certainly to the instantaneous fluctuations neglected in the theory.

**Figure 10:**
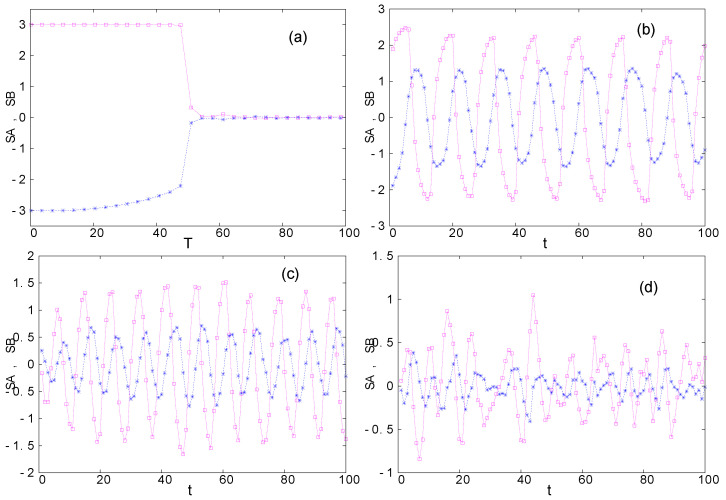
Without inter-group interactions, groups *A* and *B* with $J_{A}=J_{B}=0.02$, $q_{A}=7$, $q_{B}=7$ and initial conditions $S_{A}=-S_{B}=3$, are shown in Figure 9a with $T_{c}^{0}\left(A,B\right)=102$. The present figure shows the effect of the asymmetric interactions $K_{AB}=-0.05$, $K_{BA}=0.005$: (**a**) Under inter-group interactions, $S_{A}$ and $S_{B}$ show a critical temperature at $T_{c}=51$. (**b**,**c**) the time dependence of $S_{A}$ and $S_{B}$ at T=81 and T=115, respectively, between $T_{c}$ and $T_{c}^{0}$. Oscillations are observed. (**d**) The time dependence of $S_{A}$ and $S_{B}$ at T=166 above $T_{c}^{0}\left(A,B\right)$. Chaotic behavior is seen. See text for comments.

The same dynamics is observed when we change the inter-group interaction strength, for example, $K_{AB}=-0.05$, $K_{BA}=0.005$. However, we do not have the symmetry in their fluctuation amplitudes as seen in Figure 10.

The reader is referred to the results for other particular regions of parameters in Reference [18].

Let us show now some results for 3-group conflicts. The simulations have been carried out in the same manner as for 2-group conflicts.

**Figure 11:**
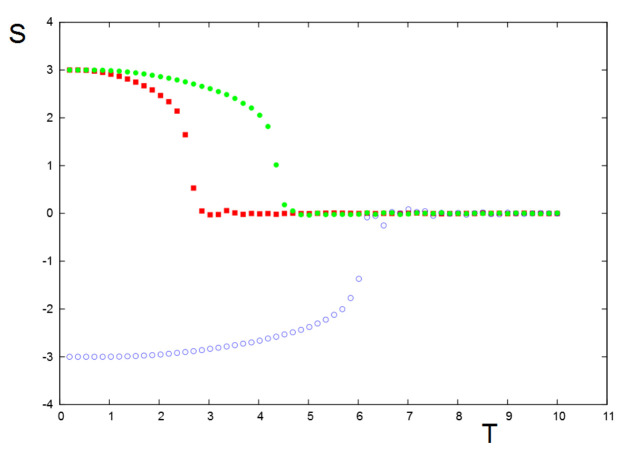
Stances of the 3 groups versus social temperature *T* in the absence of inter-group interactions. $J_{1}=0.15$, $J_{2}=0.35$, $J_{3}=0.25$. Groups 1, 2, and 3 are represented by red, blue, and green symbols, respectively. See text for comments.

We show an example in Figure 11 without inter-group interactions. For each group, there is a social temperature $T_{c}$ beyond which the stance of a group is lost, namely $S_{n}=0$. This critical social temperature is proportional to the intra-group interaction *J*: the higher *J*, the higher $T_{c}$.

As in the mean-field calculation above, an individual in a given group interacts at time t+1 with the average of the action field created by the other groups at the earlier time *t*. The only difference from the mean-field calculation is the short-range interaction considered in the Monte Carlo simulation. We will see that the results differ in some important aspects.

Once the equilibrium is reached for each group, we turn on the interactions between groups at time *t*.

**Figure 12:**
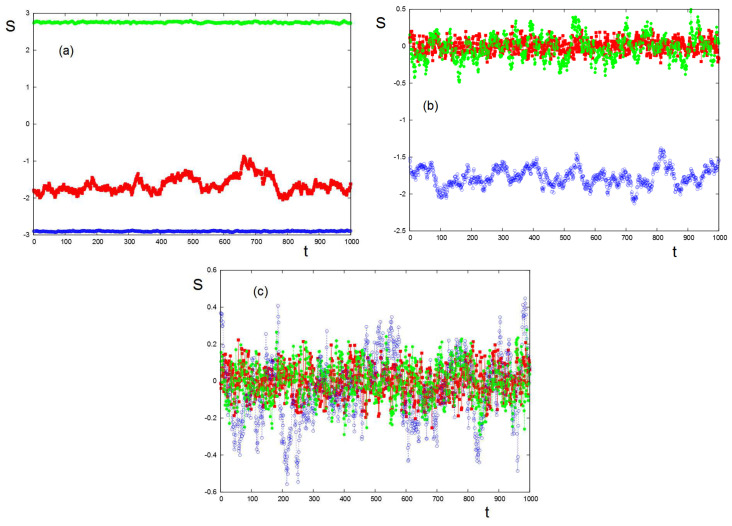
Time dependence of 3 groups’ stances at low temperatures: Groups 1, 2, and 3 are represented by red, blue, and green symbols, respectively. Inter-group interactions: $K_{12}=-0.20$, $K_{21}=0.20$, $K_{13}=-0.15$, $K_{31}=0.15$, $K_{23}=0.10$, and $K_{32}=0.10$. (**a**) T=2.5254, all three groups are ordered; (**b**) T=5.8474, groups 1 and 3 are disordered and group 2 is not disordered; and (**c**) T=7.5084, all 3 groups are disordered. The same intra-group parameters as in Figure 11 have been used: $J_{1}=0.15$, $J_{2}=0.35$, and $J_{3}=0.25$.

An example at low *T* is shown in Figure 12 where the inter-group interactions may or not destroy the order of a group. We have chosen the interaction strengths and signs in the example below to illustrate the case of Serbia–Herzegovina–Croatia 2018 election [22].

At higher *T*, the order of each group is weakened. The inter-group interactions cause the groups’ stances to oscillate widely without periodicity as also seen in the long-range mean-field results above. We observe that, at times, the stronger group 2 dominates the other two. This pattern reflects the level of intractability of the three-group conflict simulated here, consistent with the longer-term mean-field results. While the conflict is intractable at all the temperatures of Figure 12, at the lower temperature (corresponding to a stable context), the groups are “stuck” in predictable ways (see Figure 12a); as the context gets heated, the three-group system cycles unpredictably through various stages (Figure 12b,c).

These results have been discussed in the case of the Serbia–Herzegovina–Croatia election in 2018. The parameters used in Figure 12 correspond to the situation of this three-group competition [22].

## 5. Concluding Remarks

We have proposed to apply a model drawn from statistical physics to describe the trajectories in time of social conflicts among two and three groups. Interesting issues emerge from the qualitative difference between social groups and physical systems. Due to human agency, absent in physical systems, the matrix of interactions $K_{ij}$ is not symmetrical. What is the Lyapunov function for such a problem? This will be the focus of future research. We plan to expand this model in several directions that can enhance the realism of its outcomes and its applicability to a variety of social conflict situations. We will study conflicts between more than three groups by generalizing our model to multiplex networks. We will explore effects of the network topology on the qualitative behavior of this model. We will consider interactions that diminish with (social or geographic) distance between individuals. We are also interested in allowing the interactions to be determined endogenously. For example, the intra-group interactions may increase when the dynamics moves towards conflict and may decrease when the dynamics evolves away from conflict toward stalemate or even settlement. In its current version, our model is symmetric under the change of the attitude from *S* to −S, i.e., it is symmetric between the conflict and conciliatory attitudes. We intend to explore the effect of breaking this symmetry and of considering groups with different ranges of attitudes. Increasing the number of groups in the model leads to the emergence of new findings. For example, the mean-field interactions did not show chaotic behavior in the two-group case. In the case of three groups, for certain parameter choices, we find chaotic behavior (sensitivity to initial conditions, see Figure 5) and an associated strange attractor (Figure 6) that we would like to study quantitatively. If the intra-group interactions are short ranged, chaotic dynamics is exhibited even for the two-group case. We plan to refine and continue to develop the multiple group model. We are considering the introduction of principal-agent effects as we add a layer of negotiators to the layers of the disputing groups. We will explore leadership effects as well as situations where an entire group takes the position of its leader (as in dictatorships) as opposed to groups that may diverge from their leaders’ positions (as in democracies) resulting in strong or impaired cohesion levels between group members and their respective leaders. We also plan to examine sparse networks where not every individual interacts with every other in the group.
